# Laryngeal leiomyosarcoma: A rare case report and literature review

**DOI:** 10.18632/oncotarget.28862

**Published:** 2026-05-04

**Authors:** Bolat Shalabaev, Svetlana Kurash, Aibar Nurzhanov, Dulat Serikbaiuly, Bayram Kochiev, Almira Manatova, Zhandos Burkitbayev, Zhuldyz Kuanysh

**Affiliations:** ^1^Multidisciplinary Surgery Center, National Research Oncology Center, Astana, Kazakhstan; ^2^Pathomorphology Center with Cytology, National Research Oncology Center, Astana, Kazakhstan; ^3^Radiation Diagnostic Center, National Research Oncology Center, Astana, Kazakhstan; ^4^Department of Science, National Research Oncology Center, Astana, Kazakhstan; ^5^Chairman of the Board, National Research Oncology Center, Astana, Kazakhstan

**Keywords:** laryngeal leiomyosarcoma, immunohistochemistry, supraglottic mass, laryngectomy, leiomyosarcoma

## Abstract

Background: Laryngeal leiomyosarcoma (LLMS) is an exceptionally rare malignant tumor, accounting for less than 1 % of all laryngeal cancers, which are predominantly epithelial in origin. Since the first description in 1939, fewer than 70 cases have been reported worldwide. In the largest pooled analysis reported a 5-year overall survival rate of 64%, with distant metastasis identified as the main adverse prognostic factor.

Case presentation: We report a 64-year-old male who presented with progressive dyspnea and hoarseness caused by a supraglottic mass. Preoperative biopsy revealed spindle-cell proliferation consistent with leiomyosarcoma (SMA +, Vimentin +, Ki-67 60 %). Comprehensive staging with CT, MRI, and ultrasound excluded regional and distant metastases. The patient underwent extended laryngectomy with R0 margins and left neck dissection (levels II–IV). Based on multidisciplinary tumor-board discussion, four cycles of adjuvant doxorubicin and ifosfamide were administered according to national sarcoma guidelines. At 12-month follow-up, the patient remains alive and free of disease.

Conclusions: This report, representing the first documented case of LLMS from Central Asia, contributes to the limited global experience with this rare tumor. Our review identified four additional LLMS cases published between 2021 and 2024, totaling five recent reports including the present case. Collectively, these demonstrate persistent male predominance, glottic and supraglottic predilection, and survival outcomes consistent with previous observations. Complete surgical excision remains the cornerstone of therapy, while multidisciplinary-guided adjuvant treatment may benefit selected high-grade or high-risk patients. Continued accumulation and molecular characterization of cases are needed to refine prognostic assessment and optimize management strategies.

## INTRODUCTION

Leiomyosarcoma of the larynx (LLMS) is a malignant mesenchymal neoplasm of smooth muscle origin, representing <1% of laryngeal cancers [1–5]. The overwhelming majority of laryngeal malignancies are squamous cell carcinomas, whereas sarcomas such as chondrosarcoma, rhabdomyosarcoma, and angiosarcoma are exceedingly rare [4, 6–8]. The first case was described in 1939 by Jackson, and approximately 70 cases have since been reported [1–3, 5]. LLMS most likely originates from smooth muscle cells of blood vessel walls or the posterior tracheal wall [8]. Diagnosis requires histopathological evaluation supported by immunohistochemical (IHC) confirmation of smooth muscle differentiation [9, 10].

The cornerstone of treatment for laryngeal leiomyosarcoma is wide surgical excision with histologically clear margins. Chemotherapy and radiotherapy have generally limited roles but may serve as adjuncts in selected cases [11–14]. In a comprehensive review, Jin et al. (2022) analyzed 62 cases reported in the English-language literature between 1947 and 2021 [15]. Since that publication, only a few additional LLMS cases have been documented, providing new insights into surgical approaches, molecular characteristics, and long-term outcomes. In the present report, we describe a high-grade pleomorphic LLMS successfully managed with combined surgery and adjuvant chemotherapy, and we provide an updated summary of post-2022 cases to illustrate the evolving understanding and management of this rare malignancy.

## CASE REPORT

Patient, a 64-year-old male, was admitted to the National Research Oncology Center (Astana, Kazakhstan) with complaints of dyspnea and hoarseness persisting for approximately two months. On May 11, 2023, an emergency tracheostomy was performed due to acute respiratory distress caused by laryngeal stenosis. The patient was a retired school teacher. His family history was negative for malignant diseases. Relevant comorbidities included arterial hypertension, for which he was under regular follow-up by a cardiologist, and chronic pancreatitis. No other significant medical conditions were reported.

### Clinical examination

The neck’s external appearance was unremarkable, with a tracheostomy cannula in place. Indirect laryngoscopy revealed a nearly occlusive tumor with an irregular, smooth, reddish surface in the supraglottic space, along with severe edema of the left arytenoid cartilage. The vocal folds were not visible, and there was no air passage through the natural airways.

### Diagnostic assessment

**Figure 1:**
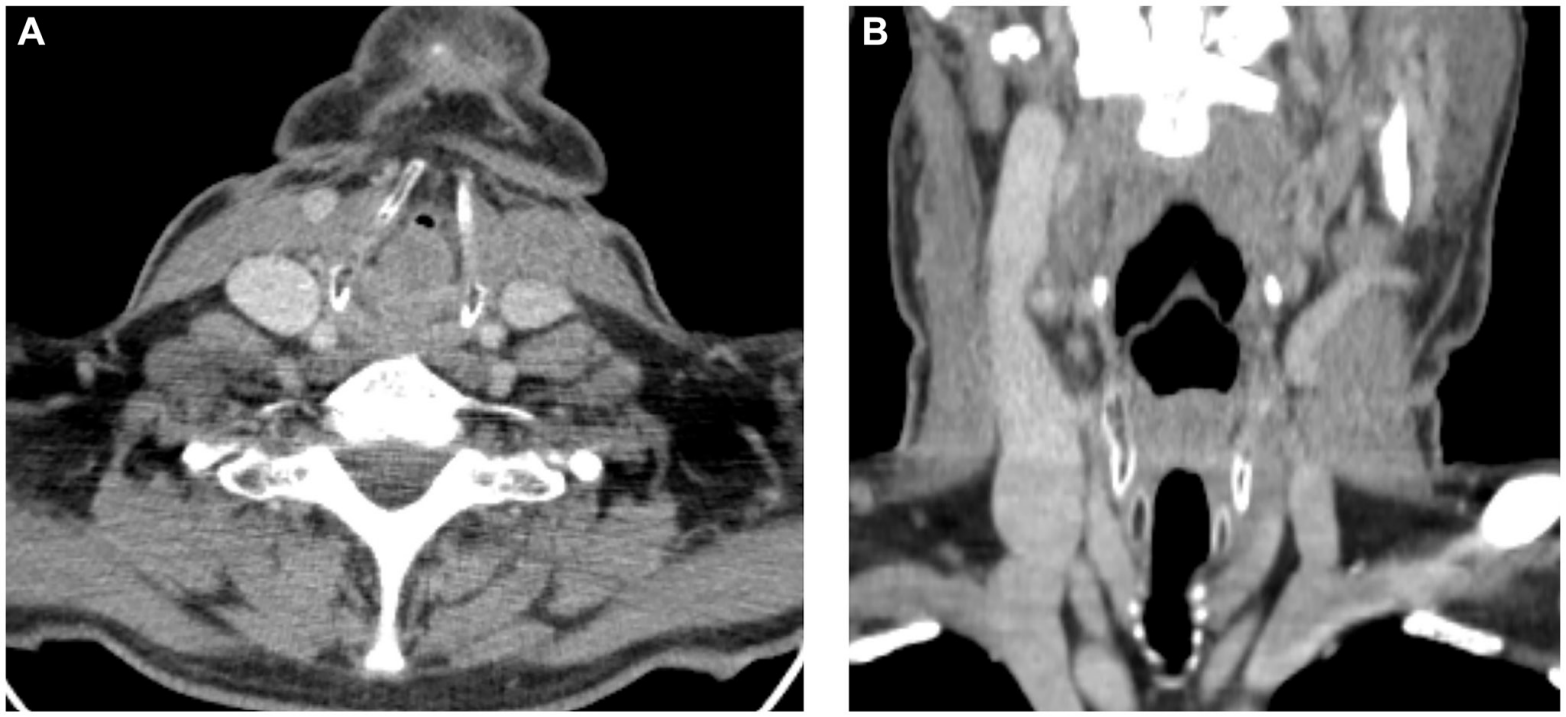
CT images in the axial (**A**) and coronal (**B**) planes demonstrate a tumor formation of the larynx, subtotally filling the lumen of the larynx.

A computed tomography examination revealed a round tumor formation, heterogeneous structure, without clear contours, with signs of invasion into the structures of the larynx, intimately adjacent to the vocal folds and subtotally narrowing the lumen of the larynx (Figure 1).

Flexible endoscopy under topical anesthesia with 10% lidocaine revealed a supraglottic mass occupying the left side of the laryngeal lumen. Biopsy samples were obtained using laryngeal forceps; minor bleeding occurred and resolved spontaneously.

Histopathological examination of the preoperative biopsy demonstrated tumor growth composed of atypical spindle cells with nuclear pleomorphism, hyperchromasia, and pathological mitoses, accompanied by sinusoid-like vascular structures and focal ulceration. Immunohistochemistry showed strong diffuse expression of smooth muscle actin (SMA) and vimentin, with a mitotic index of 50–60%, and absence of staining for CD34, myogenin, cytokeratin 5/6 and 7, and p40. These findings were consistent with a diagnosis of high-grade leiomyosarcoma (G3).

### Therapeutic intervention

On June 1, 2023 the patient underwent extended laryngectomy with formation of a persistent cannula-free tracheostomy and modified left lateral cervical lymphadenectomy (levels II–IV). Although nodal metastasis is uncommon in head-and-neck leiomyosarcomas, neck dissection was performed to ensure complete resection and minimize the risk of local recurrence [2].

The postoperative course was uneventful, and the patient was discharged in satisfactory condition on postoperative day 12.

### Histopathological and immunohistochemical findings

**Figure 2:**
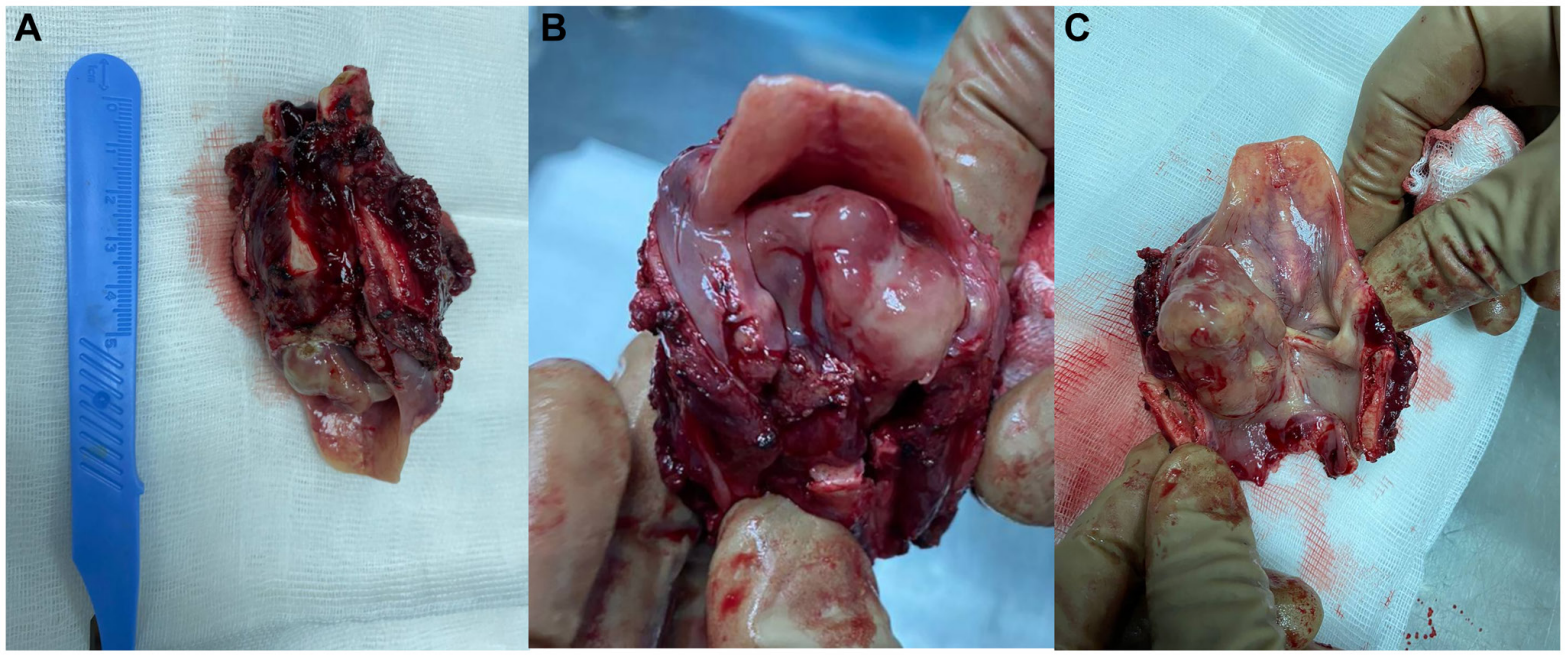
Macroscopic preparation of the larynx. (**A**) general view, (**B**) folded view, (**C**) expanded view.

The resected macropreparation was examined by histological and immunohistochemical methods. Macropreparation Figure 2A–2C - larynx measuring 8.8 × 5 × 3.5 cm, the outer surface with tissue scraps, the upper and lower edges of the resection are visually unchanged. On the section in the projection of the left vocal cord there was a tumor formation, almost completely blocking the lumen of the larynx, measuring 4.5 × 2.8 × 2.5 cm, on a wide base, the surface is gray, bumpy in places, on the sections there is an overgrowth of whitish-gray tissue with cyanotic areas, without visible growth of the thyroid cartilage. The rest of the larynx is normal. In the removed left lateral tissue of the neck, 5 lymph nodes were found.

The postoperative histology and immunoprofile fully confirmed the preoperative biopsy results, with additional extended marker evaluation yielding identical findings.

**Figure 3:**
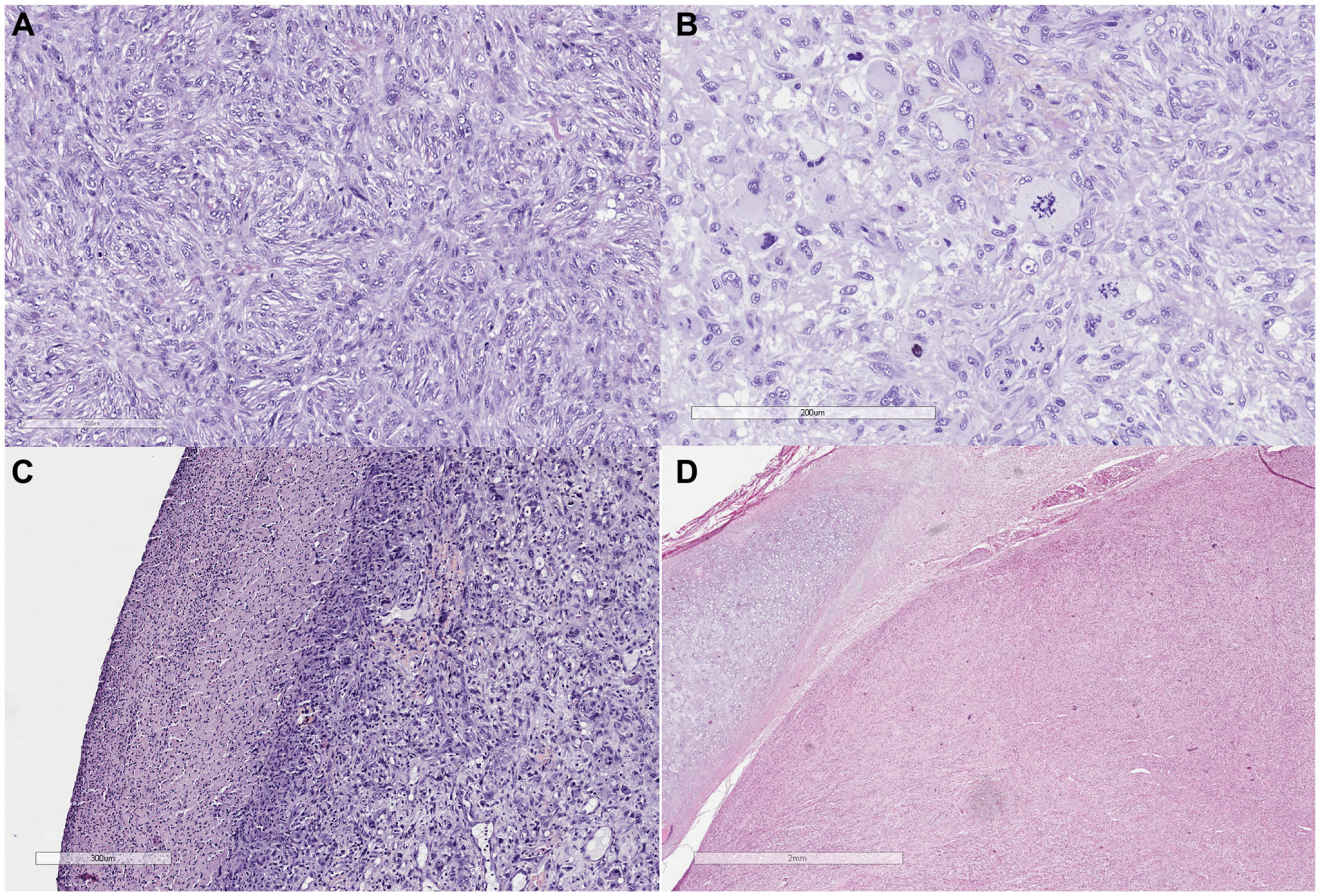
(**A**) Tumor growth is represented by the proliferation of cellular intersecting bundles of spindle-shaped cells with bright eosinophilic cytoplasm and elongated blunt-ended (cigar-shaped) nuclei, in places with the formation of a moire pattern (HE, x20). (**B**) Cell pleomorphism, pathological mitoses (HEx40). (**C**) Tumor surface with ulceration (HE, x20). (**D**) Tumor growth at the border with the laryngeal cartilage (HE, x20).

Histologically, the tumor is represented by the proliferation of cellular intersecting bundles of spindle-shaped cells with bright eosinophilic cytoplasm and elongated blunt-ended (cigar-shaped) nuclei, in places with the formation of a moire pattern; over a significant area, cells of various sizes and shapes, some pleomorphic multinucleated giants with sharply atypical nuclei. Quite numerous small vessels are visible in the tumor tissue, like sinusoids, scattered inflammatory infiltration is observed. On the surface, the formation is partially covered with multilayered squamous epithelium with preserved stratification of layers, there are large areas of ulceration with hemorrhages, inflammatory infiltration, foci of tumor tissue necrosis are visible. In the deep sections, tumor growth extended to a greater extent into the submucosa, in some fragments, ingrowth into the laryngeal muscles was observed, focal tumor tissue enveloped and focally invaded the perichondrium of the small cartilage. Tumor growth did not involve large cartilages - thyroid and cricoid, focally approached large hyaline cartilage, without growing into it. Resection margins were negative (Figure 3A–3D).

No metastatic tumor growth was detected in the five lymph nodes found.

**Figure 4:**
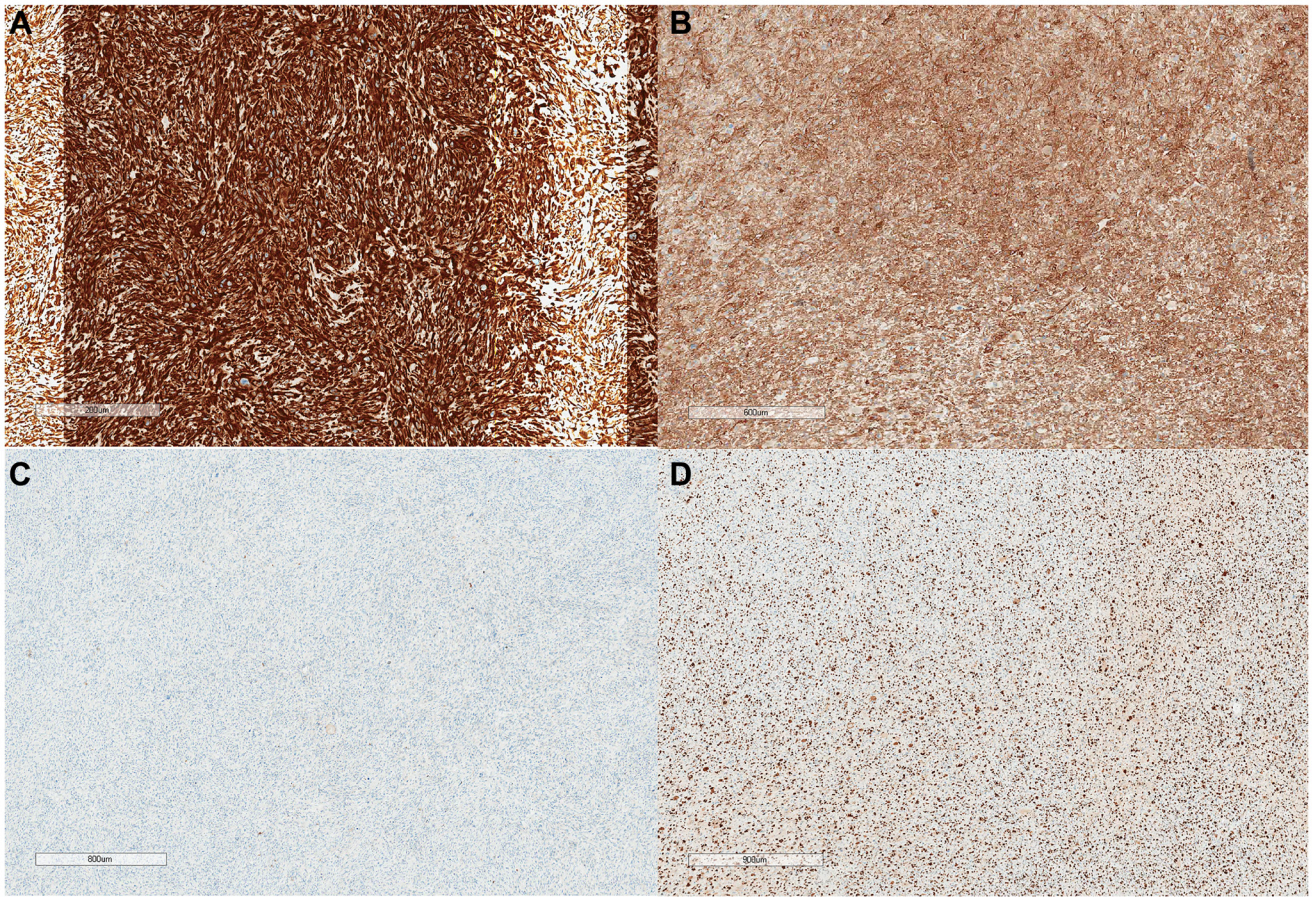
IHC study x20. (**A**) Vimentin - strong diffuse cytoplasmic and membrane expression in tumor tissue. (**B**) SMA - strong diffuse cytoplasmic expression in tumor tissue. (**C**) EMA is negative in tumor tissue. (**D**) Ki67 up to 60% in hot spots.

To verify the histogenesis of the tumor, an IHC study was performed: expression of SMA and vimentin, a high mitotic activity index of up to 60%, and no staining of tumor tissue for markers such as CD34, Myogenin, epithelial membrane antigen (EMA), transcription factor p40, cytokeratins 7 and 5/6 were detected (Figure 4A–4D).

Vimentin staining indicated a mesenchymal origin of the tumor, based on strong diffuse expression of smooth muscle actin, in the absence of staining of vascular and epithelial markers, in combination with a high proliferative activity index and a characteristic morphological picture, a diagnosis of pleomorphic leiomyosarcoma G3 was made, ICD-O code: 8890/3. According to the 8th edition AJCC TNM classification, the stage was pT3N0M0.

### Treatment rationale and follow-up protocol

To exclude distant metastases prior to treatment initiation, the patient underwent a comprehensive staging work-up that included contrast-enhanced computed tomography (CT) of the chest, abdomen, and pelvis; magnetic resonance imaging (MRI) of the head and neck; and abdominal ultrasonography. No evidence of regional or distant metastatic disease was detected, consistent with a localized primary laryngeal lesion, corresponding to an M0 category.

All investigations were performed in accordance with the national soft-tissue sarcoma diagnostic protocol adopted by the Ministry of Health of the Republic of Kazakhstan (MoH RK, 2023) [16].

The decision to initiate adjuvant chemotherapy was made following a multidisciplinary tumor board discussion that included head and neck surgeons, pathologists, oncologists, and radiologists. The tumor’s high-grade morphology, marked nuclear pleomorphism, and Ki-67 proliferation index of 60% placed the patient in a high-risk group for early recurrence or micrometastatic dissemination. Based on these histologic features and the recommendations of the national clinical guidelines for soft-tissue sarcomas (MoH RK, 2023), adjuvant chemotherapy (4–6 courses) with doxorubicin plus ifosfamide was selected. This regimen is considered the standard first-line combination for aggressive or disseminated soft-tissue sarcomas, aiming to eradicate potential micrometastases and reduce the likelihood of relapse in patients with high-grade disease.

Post-treatment surveillance was organized according to the National follow-up protocol for soft-tissue sarcomas, which mandates regular oncologic supervision at the patient’s local oncology center. Follow-up visits are scheduled every 3 months during the first two years, every 6 months during the third year, and annually thereafter from the fourth year onward. The standard follow-up imaging includes CT of the primary tumor region, CT of the abdomen and pelvis, and CT or PET/CT of the chest to monitor for local recurrence or distant metastasis.

At the most recent follow-up, 12 months after surgery and completion of adjuvant chemotherapy, the patient remained alive and free of disease recurrence or metastasis.

## DISCUSSION

Laryngeal leiomyosarcoma remains an extraordinarily rare entity, with limited data guiding treatment. The recent review by Jin et al. (2022) compiled 62 English-language cases, identifying a strong male predominance, a mean age of 59 years, and glottic involvement in over two-thirds of patients [15]. Since that report, five additional cases of LLMS have been identified in the English- and Russian-language literature, including the present case (Table 1) [14, 17–19]. To identify newly published cases of laryngeal, a targeted literature search was conducted in PubMed and Scopus databases covering the period from January 2021 to January 2025.

**Table 1:** Summary of post-2021 laryngeal leiomyosarcoma cases

**Author (Year)**	**Age/Sex**	**Site**	**IHC profile**	**Treatment**	**Margins**	**Adjuvant therapy**	**Outcome/Follow-up**
Allen et al., 2023 [14]	40/M	Right larynx, hypopharynx, right thyroid cartilage	SMA + (α-smooth muscle actin), G2/3	Partial laryngectomy, pharyngectomy, neck dissection	R0	60 Gy RT	No recurrence at 3 months
Ugrinović et al., 2021 [17]	58/M	Ventricular, vocal folds	ASMA +, Calponin +, h-Caldesmon +, Ki-67 40%	Total laryngectomy	R0	None	Recurrence, lung metastasis, death 6 months after laryngectomy
Kozhanov et al., 2024 [18]	48/M	Right vocal fold, subglottis	SMA +, G1–2	Total laryngectomy, voice prosthesis	R0	None	Alive, no recurrence 16 yr
Mignogna et al., 2023 [19]	67/M	Glottis	MYC, FGFR1, AURKA, PTEN, TP53 mutations	Total laryngectomy, bilateral neck dissection	R0	None	Not stated
Present case (NROC, 2025)	64/M	Supraglottis (left vocal fold)	SMA +, Vimentin +, CD34 –, Myogenin –, EMA –, p40/CK7/CK5-6 –, Ki-67 60 %	Extended laryngectomy, left neck dissection (II–IV)	R0	4 cycles Doxorubicin + Ifosfamide	Alive, no recurrence 12 months

Abbreviations: SMA: smooth muscle actin; ASMA: α-smooth muscle actin; IHC: immunohistochemistry; RT: radiotherapy; R0: negative surgical margins (no residual tumor); EMA: epithelial membrane antigen; CK: cytokeratin; Ki-67: proliferation index; CD34: cluster of differentiation 34; MYC: MYC proto-oncogene; FGFR1: fibroblast growth factor receptor 1; AURKA: aurora kinase A; PTEN: phosphatase and tensin homolog; TP53: tumor protein p53; h-Caldesmon: high-molecular-weight caldesmon; G1–3: tumor grade 1–3; +: positive; –: negative.

Among these five patients, all were male, and the age at diagnosis ranged from 40 to 67 years (mean, 55.4 years). The majority of tumors (3 of 5, 60%) originated from or involved the glottic and supraglottic regions, while one case each arose from the hypopharyngeal-laryngeal junction and the ventricular fold.

The clinical presentation was dominated by hoarseness (4 of 5, 80%), followed by dyspnea or airway obstruction and dysphagia. One patient required emergency tracheostomy due to critical laryngeal stenosis. Imaging modalities such as CT or MRI consistently revealed well-circumscribed or submucosal masses with variable enhancement patterns, similar to those previously described by Jin et al. (2022) [15].

Differential diagnosis includes spindle-cell squamous carcinoma, rhabdomyosarcoma, and malignant peripheral nerve sheath tumor. IHC remains essential: SMA, desmin, and h-caldesmon positivity, alongside negativity for epithelial (AE1/AE3, p40), neural (S100, SOX10), and vascular (CD34) markers, confirms smooth muscle origin [14, 20]. EBV association, rare and typically seen in immunosuppressed patients, was not identified in any recent post-2021 LLMS reports. These findings are consistent with previous reports, which emphasize the utility of immunohistochemistry in differentiating leiomyosarcoma from other spindle cell neoplasms such as fibrosarcoma, rhabdomyosarcoma, and malignant peripheral nerve sheath tumors [20, 21].

Comparatively, the prognosis of laryngeal leiomyosarcoma remains variable and generally poor, largely due to its aggressive behavior and the potential for local recurrence and distant metastasis. In our case, the achievement of clear surgical margins were favorable prognostic factors. This outcome aligns with the findings of studies by Eldeeb et al. and Patel et al. which reported better survival rates in patients with negative surgical margins [22, 23]. However, the high-grade nature of the tumor, indicated by a high mitotic index and pleomorphism, necessitated adjuvant chemotherapy, a treatment approach that remains controversial in the literature.

The largest pooled analysis of LLMS by Jin et al. (2022) reported an overall 5-year survival rate of 64%, with no statistically significant differences in survival according to sex, age, site, or treatment modality. However, distant metastasis emerged as the only variable significantly influencing survival (*p* = 0.040), highlighting the need for vigilant long-term monitoring and aggressive management of high-risk disease. Our review adds four additional post-2021 LLMS cases, together with the present case, expanding the cumulative literature. Among these recently reported patients, survival outcomes remain heterogeneous: one patient died within six months due to pulmonary metastasis, one remained disease-free at 3 months, one at 16 years, and one case had no outcome reported. The current patient is alive and recurrence-free at 12 months of follow-up. Taken together, these results illustrate the marked variability in clinical course even among patients with complete (R0) resections and confirm that long-term surveillance is essential, as late recurrences may occur years after initial treatment.

The review by Jin et al. demonstrated that surgical resection remains the cornerstone of treatment for laryngeal leiomyosarcoma. Total laryngectomy was the most frequently performed procedure, although partial laryngectomy and transoral CO_2_ laser excision were commonly used for early-stage glottic tumors. Importantly, long-term survival was associated primarily with complete tumor excision with negative margins rather than with the extent of surgery. Adjuvant radiotherapy was applied selectively in patients with high-grade tumors or close/positive margins, whereas systemic chemotherapy was rarely used and generally reserved for locally advanced or unresectable disease, with limited impact on survival. The longest-surviving patients in the Jin et al. cohort were those who achieved complete surgical resection, either alone or combined with risk-adapted radiotherapy. These findings suggest a gradual paradigm shift toward individualized, multidisciplinary treatment strategies that prioritize oncologic clearance while balancing functional preservation [15].

While adjuvant chemotherapy is not uniformly recommended due to its limited efficacy in leiomyosarcomas, our patient underwent four courses of doxorubicin and ifosfamide. This regimen was chosen based on its documented activity against high-grade sarcomas and National clinical protocol, despite mixed evidence regarding its impact on overall survival. The decision to administer chemotherapy was also influenced by the tumor’s poor differentiation and high proliferative activity, factors associated with a higher risk of recurrence. In contrast, other studies, such as those by Ajithkumar et al., suggest that surgery alone may suffice for localized, low-grade tumors, emphasizing the role of individualized treatment planning based on tumor characteristics [24].

This report has several limitations inherent to both the single-case nature of the presentation and the available literature. First, the rarity of laryngeal leiomyosarcoma and the small number of recently reported cases limit the ability to draw definitive conclusions regarding optimal treatment strategies or prognostic factors. Second, although the postoperative outcome is favorable, the follow-up period remains relatively short, and late recurrence or distant metastasis cannot be fully excluded. Third, molecular profiling was not uniformly available across all reviewed cases, restricting comparative analysis of genomic alterations. In addition, endoscopic images from the initial diagnostic evaluation were not archived, representing a technical limitation of this case; however, the diagnosis was supported by concordant preoperative biopsy and postoperative histopathological and immunohistochemical findings. Despite these constraints, detailed documentation of clinical presentation, treatment, and outcome contributes meaningful data to the limited evidence base on this rare malignancy.

## CONCLUSIONS

This case and the five most recent reports confirm that laryngeal leiomyosarcoma is a highly heterogeneous and unpredictable malignancy. Beyond its rarity, the present case is notable for its high-grade histology, comprehensive preoperative diagnostic work-up, and multidisciplinary treatment approach guided by national sarcoma protocols. Our experience supports the central role of complete surgical excision with histologically negative margins and the selective use of adjuvant chemotherapy in patients with high-risk pathological features. Furthermore, this report represents the first documented case of laryngeal leiomyosarcoma from Central Asia, expanding the geographic and clinical spectrum of the disease. Continued reporting of well-characterized cases, including treatment details and follow-up outcomes, is essential for improving prognostic stratification and progressing toward evidence-based management strategies for this rare entity.
